# Deep Learning-Based Morphological Classification of Endoplasmic Reticulum Under Stress

**DOI:** 10.3389/fcell.2021.767866

**Published:** 2022-01-21

**Authors:** Yuanhao Guo, Di Shen, Yanfeng Zhou, Yutong Yang, Jinzhao Liang, Yating Zhou, Ningning Li, Yu Liu, Ge Yang, Wenjing Li

**Affiliations:** ^1^ Laboratory of Computational Biology and Machine Intelligence, National Laboratory of Pattern Recognition, Institute of Automation, Chinese Academy of Sciences, Beijing, China; ^2^ School of Artificial Intelligence, University of Chinese Academy of Sciences, Beijing, China; ^3^ CAS Key Laboratory of Separation Science for Analytical Chemistry, Dalian Institute of Chemical Physics, Chinese Academy of Sciences, Dalian, China; ^4^ Tomas Lindahl Laboratory, The Seventh Affiliated Hospital, Sun Yat-Sen University, Shenzhen, China

**Keywords:** ER stress, morphological classification, image biomarker, deep learning, homeostasis

## Abstract

Endoplasmic reticulum stress (ER stress) is a condition that is defined by abnormal accumulation of unfolded proteins. It plays an important role in maintaining cellular protein, lipid, and ion homeostasis. By triggering the unfolded protein response (UPR) under ER stress, cells restore homeostasis or undergo apoptosis. Chronic ER stress is implicated in many human diseases. Despite extensive studies on related signaling mechanisms, reliable image biomarkers for ER stress remain lacking. To address this deficiency, we have validated a morphological image biomarker for ER stress and have developed a deep learning-based assay to enable automated detection and analysis of this marker for screening studies. Specifically, ER under stress exhibits abnormal morphological patterns that feature ring-shaped structures called whorls (WHs). Using a highly specific chemical probe for unfolded and aggregated proteins, we find that formation of ER whorls is specifically associated with the accumulation of the unfolded and aggregated proteins. This confirms that ER whorls can be used as an image biomarker for ER stress. To this end, we have developed ER-WHs-Analyzer, a deep learning-based image analysis assay that automatically recognizes and localizes ER whorls similarly as human experts. It does not require laborious manual annotation of ER whorls for training of deep learning models. Importantly, it reliably classifies different patterns of ER whorls induced by different ER stress drugs. Overall, our study provides mechanistic insights into morphological patterns of ER under stress as well as an image biomarker assay for screening studies to dissect related disease mechanisms and to accelerate related drug discoveries. It demonstrates the effectiveness of deep learning in recognizing and understanding complex morphological phenotypes of ER.

## Introduction

The endoplasmic reticulum (ER) is a continuous membrane-bound organelle network that plays key roles in protein synthesis and modification, lipid biogenesis, and ionic homeostasis (Friedman and Voeltz, 2011). Dysregulation of protein synthesis and modification caused by intra- and extra-cellular cues leads to excessive accumulation of unfolded proteins, which triggers ER stress (Lee et al., 2015; Schwarz and Blower, 2016). Unfolded protein response (UPR) often refers to the signal transduction pathway for cellular response to ER stress (Hetz et al., 2020). There are three main UPR transducers: inositol-requiring enzyme 1 (IRE1) (Tirasophon et al., 1998; Wang et al., 1998), protein kinase RNA-like ER kinase (PERK) (Harding et al., 1999), and activating transcription factor 6 (ATF6) (Harding et al., 2000; Ron and Walter, 2007). By orchestrating cellular processes such as mRNA splicing by endoribonuclease IRE1α (Tirasophon et al., 1998; Wang et al., 1998), translation attenuation by kinases PERK (Harding et al., 1999), and protein folding assistance by chaperone BiP (binding immunoglobulin protein) (Shimizu et al., 2017), the UPR engages different outputs to restore ER protein homeostasis under mild stress conditions or to activate apoptosis under chronic stress conditions (Yoon et al., 2012; Nie et al., 2017; Ramachandran et al., 2021).

ER stress is strongly implicated in the onset and progression of a wide range of human diseases, including neurodegenerative diseases, metabolic diseases, and cancer (Ozcan et al., 2004; Zhang et al., 2005; Wang and Kaufman, 2016). It can cause not only alterations of protein synthesis or folding but also deleterious cellular responses including accumulation of lipids and activation of autophagy. Modulating ER stress shows great potential in the treatment of these diseases. Several compounds, including IRE1 α inhibitor KIRA6 and PERK inhibitor GSK2656157 (Axten et al., 2012; Wang et al., 2012; Ghosh et al., 2014), have been identified in target-based drug screening to modulate ER stress and have shown therapeutic benefits (Nunes et al., 2012; Ghosh et al., 2014; Kitakaze et al., 2019). In comparison to target-based screening, phenotypic screening utilizes readouts that are more observable and physiologically relevant for drug discoveries (Swinney and Anthony, 2011; Moffat et al., 2017). It can accelerate drug discoveries by using cell models of diseases with the support of high-throughput imaging (Zheng et al., 2013; Moffat et al., 2017). However, reliable and sensitive image biomarkers are required for phenotypic screening. Although ER morphology is a key cellular phenotypic feature, it is unclear whether it can serve as an image biomarker for ER stress. In addition, for any image biomarker, a reliable and efficient detection assay is essential for phenotypic screening (Kazama et al., 2018). Such an assay has yet to be developed for ER stress.

Changes in ER morphology correlate well with ER stress (Schuck et al., 2009; Mateus et al., 2018). The classical ER structure consists of a continuous envelope surrounding the nucleus in the perinuclear region and a polygonal network of interconnected tubules and sheets in the peripheral region (Friedman and Voeltz, 2011). In ER-stressed cells, ER membranes are compacted to form ER whorls (Nii et al., 1968; Snapp et al., 2003). Formation of ER whorls provides an effective structural response to prolonged ER stress (Xu et al., 2020). It accompanies the activation of PERK and works together with vesicle transport machinery such as ESCRT (endosomal complexes required for transport) and COPII (coat protein complex II) complex to counter-balance ER stress-induced protein translation and ER expansion (Bernales et al., 2006; Schuck et al., 2014; Schäfer et al., 2020; Xu et al., 2020). ER whorls have been observed in yeast and mammalian cells under ER stress activated by various stimuli, including drugs and herpes simplex virus infection (Nii et al., 1968; Schäfer et al., 2020). To use ER whorls as an image biomarker for phonotypic drug screening, reliable and automated detection is essential. However, detection of ER whorls within the complex and dense ER network morphology poses a substantial technical challenge (Kazama et al., 2018).

The past decade witnessed the rapid rise of deep learning as a transformative artificial intelligence technique that computes using deep neural networks (DNNs). It has achieved breakthrough performance in many challenging tasks of analyzing natural images (LeCun et al., 2015). It has also achieved breakthrough performance in analyzing cellular images that previously are considered intractable for traditional methods (Moen et al., 2019). Unlike traditional methods, which rely on manually designed features to represent phenotypes in images, deep learning models automatically learn phenotypic features through their supervised training (LeCun et al., 2015). Recently, for example, deep learning models such as ResNet (He et al., 2016) and DenseNet (Huang et al., 2017) have achieved great success in recognizing cell states (Godinez et al., 2017; Sommer et al., 2017) and protein subcellular localization patterns (Kraus et al., 2016; Pärnamaa and Parts, 2017).

In this study, by comparing ER morphology under normal conditions *versus* IRE1 α activation, we found that the formation of ER whorls is initiated specifically when UPR pathways are activated under induced ER stress and that it is dependent on the duration and strength of the induced ER stress. By using a highly specific chemical probe, we found that whorls are tightly associated with unfolded and aggregated proteins. This confirms that ER whorls can serve as an image biomarker for ER stress. To use it as an image biomarker for screening studies, we have developed a deep learning-based image analysis assay, the ER-WHs-Analyzer, that recognizes and localizes ER whorls automatically. It includes a feature recognition module that achieves over 95% accuracy in recognizing ER whorls and classifying their patterns. It also includes a feature localization module that reliably detects regions of ER whorls in a manner consistent with the visual inspection by human experts. Training of deep learning models of ER-WHs-Analyzer requires no manual annotation of precise locations of ER whorls. Through a double-blind experiment, we further confirmed that ER whorls can serve as a reliable image biomarker for ER stress. Importantly, ER-WHs-Analyzer can reliably classify different patterns of ER whorls induced by different ER stress activation reagents. Overall, our study provides mechanistic insights into the relations between unfolded and aggregated proteins and ER whorls. It also provides an image biomarker assay for automated and quantitative analysis of ER stress that is well suited for phenotypic screening for related disease mechanism studies and drug discoveries.

## Materials and Methods

### Induction and Western Blot Analysis of ER Stress in HEK293T Cells

HEK293T cells were treated with DMSO or ER stress induction compounds Thapsigargin (Tg) or Dithiothreitol (DTT) for various lengths of time based on experimental designs, typically 6 h, then trypsinized, pelleted (800 × g, 4 min, room temperature) and lysed in RIPA buffer (Millipore) with protease inhibitor (Roche) and phosphatase inhibitor on ice. The supernatant was collected by centrifugation (13,000 × g, 15 min, 4°C) and the protein concentration was determined using BCA protein assay (Beyotime). Proteins were resolved by 12% SDS-PAGE, transferred to PVDF membranes, then blocked with 5% non-fat milk in TBST for 1 h at room temperature. The membranes were then washed with TBST (3 × 10 min) and incubated with anti-IRE1 (CST), anti-p-IRE1 (Abcam), or anti-Caspase 3 (CST) overnight at 4°C. Next, the membranes were washed with TBST (3 × 10 min) and incubated with goat-anti-rabbit-HRP or goat-anti-mouse-HRP in TBST for 1 h at room temperature. After washing with TBST for 3 times, the blots were developed using an ECL detection reagent.

### Live Cell Imaging

High-resolution images of ER in live cells were acquired under two conditions, i.e., normal ER morphology in control cells and abnormal ER morphology (i.e., with ER whorls) in cells under induced ER stress. HEK293T cells labeled with ER marker GFP-sec61b were treated with DMSO, Tg or DTT for various lengths of time based on experimental designs, typically 6 h. To check abundance and location of ER proteins on whorls, BFP-KDEL was transiently expressed in HEK293T cells to label luminal proteins. And mCherry-Rtn4a, mCherry-ATL3, and GFP-REEP5 were transiently expressed in HEK293T cells to label ER morphology regulator proteins reticulon, atlastin, and REEP5, respectively. Treated cells were then imaged using conventional spinning disk confocal microscopy at ∼200 nm resolution (Nikon CSU-W1 under 100× and 1.45 NA, excitation wavelength: 488 nm, emission wavelength: 535 nm) or 3D-SIM at ∼70 nm resolution (Nikon N-SIM, 100× SR objective, excitation wavelength: 488 nm, emission wavelength: 535 nm).

### Synthesis of the AIEgen Probe for Misfolded and Aggregated Proteins

A 250 ml round bottom flask was charged with isophorone (6.0 ml, 40 mmol), malononitrile (2.9 g, 44 mol) and piperidine (cat.). The mixture was heated to reflux and stirred for 30 h. After cooling to room temperature, the solution was slowly poured into water and the precipitated solid was filtered. Recrystallization from EtOH afforded S1 as a brown solid (2.1 g, 28%). To a 25 ml round bottom flask S1 (186.3 mg, 1.0 mmol), 4-(bis(2-hydroxyethyl)amino) benzaldehyde (313.8 mg, 1.0 mmol) and piperidine (cat.) were stirred in 10 ml EtOH at 85°C for 20 h. The solution was cooled to room temperature, and water was added to the solution. Then the mixture was extracted with DCM (3 × 20 ml). The organic phase was combined and dried over Na_2_SO_4_. After filtration and concentration in vacuo, the residue was purified *via* flash silica gel chromatography (10–40% EtOAc in hexane) to provide the compound AIEgen as violet solid (231.4 mg, 61.3%) and analyzed by 1H-NMR (400 MHz, CDCl_3_).

### *In vitro* Thermal Shift Assay

AIEgen (25 μ M) and 1) WT-DHFR (50 μ M), 2) mut-DHFR (50 μ M), and 3) sortase (50 μ M) were mixed in acidic aggregation buffer (NaOAc 200 mM, KCl 100 mM, acidified by AcOH to pH = 6.23) and incubated at 60°C for 5 min 4) AIEGEN (25 μ M) and human Ig (50 μ M) were mixed in acidic aggregation buffer (NaOAc 200 mM, KCl 100 mM, acidified by AcOH to pH = 6.23) and incubated at 80°C for 5 min 5) AIEGEN (25 μ M) SOD1 (50 μ M) were mixed in buffer A (50 mM Tris-HCl, 100 mM NaCl, acidified by HCl to pH = 8.0) and EDTA disodium salt (250 mM). The mixture was incubated at 60°C for 5 min. Spectra were collected with excitation wavelength of 561 nm. All measurements were carried out using Tecan Spark fluorescence plate reader in BeyoGoldTM 96-well black opaque plates.

### ER-WHs-Analyzer

Image pre-processing and data augmentation – Each of the high-resolution ER images acquired contains multiple cells. Each single cell was first cropped from the acquired full-size images. The cropped images were resized into 256 × 256 pixels with zero padding for its shorter side to keep the original aspect ratio of the single cell. Intensity stretch and histogram equalization were applied to enhance the images. To avoid overfitting when training deep learning models, data augmentation by random flipping and rotation was performed online during training. This produced a considerably diverse data combination. Further details on the image datasets are given in the Results section.

Feature recognition module – It is the module used for recognizing and classifying ER morphological patterns, with or without ER whorls. Two types of representative DNNs, the ResNet (He et al., 2016) and DenseNet (Huang et al., 2017), were chosen as the backbone network of the feature recognition module. The recognition of ER whorls, present or absent, is a standard classification problem. The Softmax function was used to map output of the DNNs into a classification score:

$$p\left(y^{l}|x\right)=\frac{e^{-f^{l}}}{\sum_{j}e^{-f^{j}}}$$ (1)

In Eq. 1, x denotes the input image; $p\left(y^{l}|x\right)$ denotes the probability that the image x is classified as in class l ; f is the output of the DNN parameterized by f=ℱ(x;ℱ) , where ℱ is the composition of the network parameters and ℱ represents model weights; $f^{j}$ is the *j-th* element in f , which denotes the probability that image x is classified as in the *j-th* class. The following Cross-Entropy loss was used for model training:

$$ℒ=\frac{1}{N}\overset{N}{\underset{i=1}{\sum }}-y_{i}^{l}\log p\left({\hat{y}}_{i}\text{|}x_{i}\right),$$ (2)

where $y_{i}^{l}$ is the one-hot coding of the ground-truth for the *i-th* input image; the *l-th* element of $y_{i}^{l}$ takes the value of 1 for its ground-truth class, and the rest takes the value of 0; $p\left({\hat{y}}_{i}\text{|}x_{i}\right)$ represents the predictions of the network for the input image, which are normalized using Eq. 1; N is the number of training examples. The goal is to minimize the loss by training the DNNs to obtain predictions that best match the ground truth. The standard optimization method stochastic gradient descent (SGD) was used in this study.

Training strategies - Two training strategies were used: training from scratch and finetuning from a pre-trained model, i.e., transfer learning (Tan et al., 2018), a widely used approach to stabilize the training of deep learning models. To this end, a large dataset was collected primarily from open-source microscopy images, named as CBMI-Extra, which includes ∼70k images from ∼120 classes. Some of the images in CBMI-Extra were acquired using imaging protocols similar as those for the ER images in this study. In this way, a deep learning model pre-trained using CBMI-Extra provided a sound starting point to stabilize its subsequent training using ER images.

Feature localization module – A deep learning model with good performance is expected to capture image features similarly as human experts. The feature visualization tool, Grad-CAM (Selvaraju et al., 2017), was used to check whether the DNN models can correctly recognize features of the ER whorls. Given an input image for a forward pass in a trained network, the feature visualization tool generates a class activation mapping (CAM) in the form of a heatmap, visualizing the importance of each location in the input image in terms of its contribution to the prediction of the network. In this way, the feature visualization tool Grad-CAM constitutes the first part of the feature localization module by identifying up to several hotspots (i.e., clusters with higher scores) in the heatmap. It has been observed that the identified hotspots in the ER images match well with the ER morphological features that a human expert would identify. Based on the hotspots in heatmaps, an image processing-based pipeline was used to detect locations of the regions of ER whorls. This pipeline, which consists of segmentation, instance labeling and bounding-box assignment, constitutes the second part of the feature localization module (Supplementary Figure S1).

## Results

### ER Stress Induces Morphological Deformation That Forms Whorls

**FIGURE 1:**
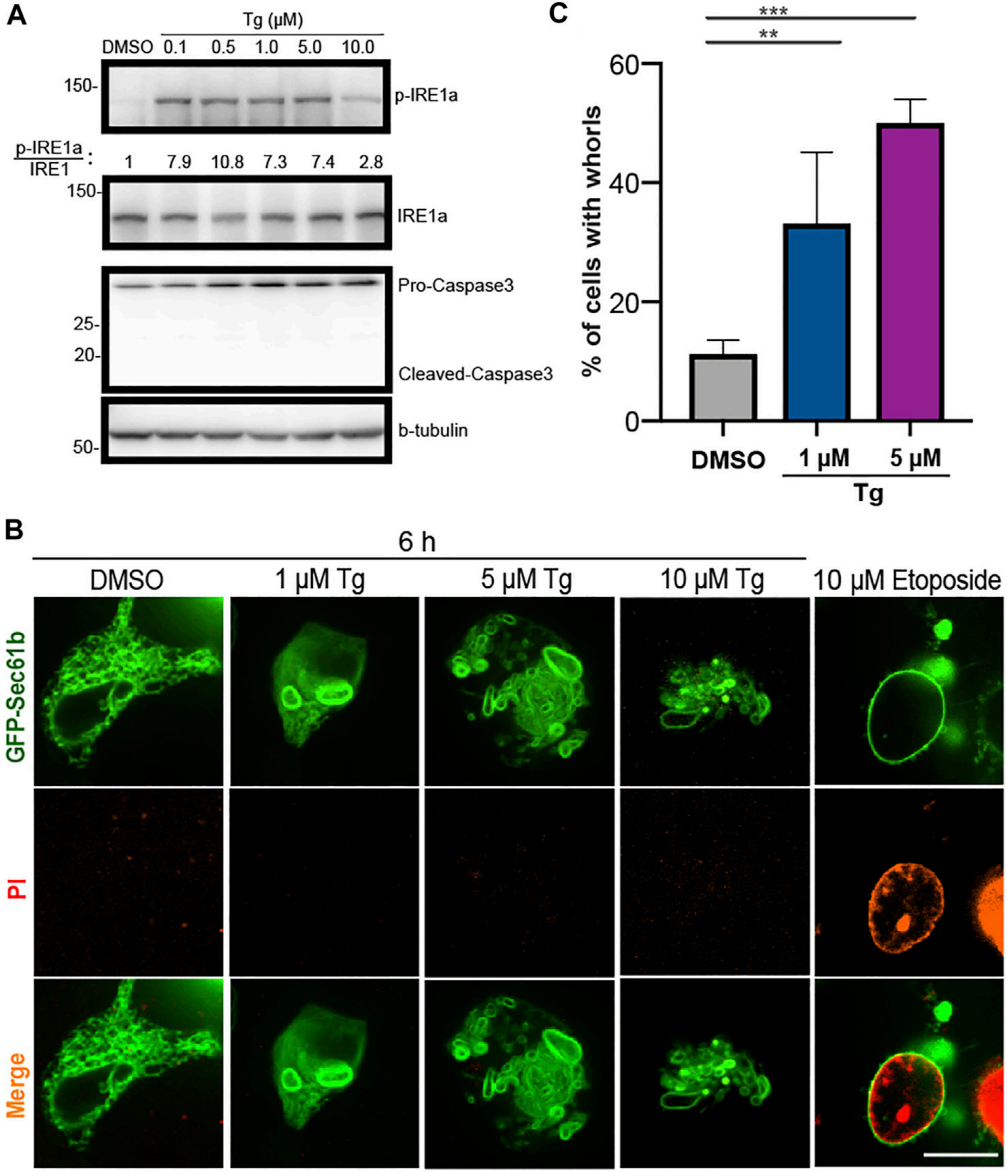
Upregulated ER stress markers and abnormal ER shapes in HEK293T cells treated with Tg at different concentrations. **(A)** Immunoblot analysis of p-IRE1 α , IRE1 α , caspase3 (pro and cleaved) and β-tubulin from cell lysates after treatment of HEK293T cells with DMSO or Tg at the indicated concentrations for 6 h. The ratio of p-IRE1 α to IRE1  α is shown for each concentration. **(B)** Representative ER structures labeled with GFP-Sec61β (green) in HEK293T cells treated with Tg at the indicated concentrations for 6 h. PI staining was used to detect cell apoptosis. No substantial PI staining signal was detected in Tg treated cells (second row). Etoposide treated cells were used as a reference and a positive control for PI staining. Scale bar: 10 μ m. **(C)** Percentage of HEK293T cells with ER whorls after treatment with DMSO or Tg at different concentrations for 6 h. Error bars indicate standard deviation (SD) calculated from three independent experiments. **: *p* < 0.01, ***: *p* < 0.001.

**FIGURE 2:**
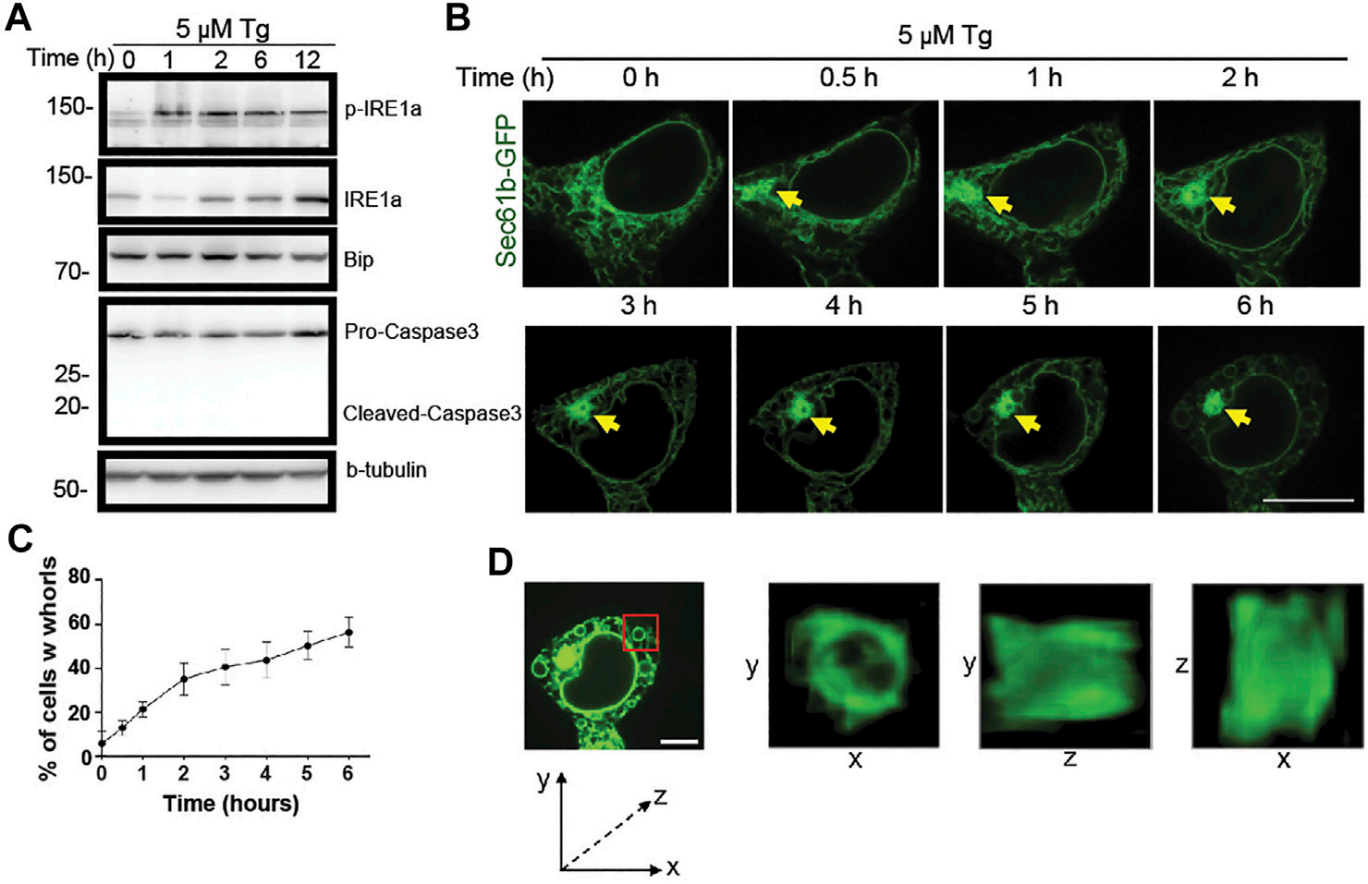
Progressive changes of ER structure under ER stress over time. **(A)** Immunoblot analysis of p-IRE1 α , IRE1 α , caspase3 (pro and cleaved) and β-tubulin from cell lysates after treatment of HEK293T cells with DMSO or 5 μ M Tg for the indicated durations. **(B)** Representative ER structures labeled with GFP-Sec61 β (green) in HEK293T cells treated with 5 μ M Tg for the indicated durations. Yellow arrows point to where initial formation and evolution of an ER whorl occur. Scale bar: 20 μ m. **(C)** Percentages of HEK293T cells with ER whorls after treatment with 5 μ M Tg for the indicated durations. Data were presented as mean ± SD from three independent experiments. **(D)** 5 × magnified 3D views of the ring-like ER whorl in the region marked by the red box in the leftmost sub-panel. Scale bar: 10 μ m.

We used ER stress activator Thapsigargin (Tg) to set up our experimental assay (Dibdiakova et al., 2019). Because Tg activation of ER stress increases the level of phosphorylated IRE1 α (Han et al., 2009), we used the amount of phosphorylated IRE1 α normalized by the total amount of IRE1α as an indicator of ER stress. We checked this indicator at different concentrations and durations of Tg treatment (Figures 1A, 2A). We found that Tg treatment can reliably activate ER stress at a concentration ranging from 0.1 to 10 μ M and a duration ranging from 6 to 12 h without affecting cell viability (Figures 1A, 2A). Based on these results, we set the concentration and duration of Tg treatment at 5 μ M and 6 h, respectively, for subsequent experiments.

To investigate whether activated ER stress causes morphological changes, ER was labeled by stable expression of GFP-Sec61 β , a subunit of the Sec61 translocon complex located on ER membrane. After activation with Tg, ER morphology in HEK293T cells was examined using spinning disk confocal microscopy at three concentrations for comparison (1, 5, 10 μ M). Consistent with Western blot analysis of IRE1 α , live-cell imaging of ER revealed morphological changes at all three concentrations (Figures 1B,C). In control cells treated with only DMSO, ER exhibited an interconnected membrane network that extends from the nuclear envelope (Figure 1B). However, under Tg treatment, ER formed multiple whorls that aggregate near the nuclear envelope (Figure 1B). Previous studies found that formation of ER whorls is a dynamic and reversible response to strong ER stress (Xu et al., 2020). Consistent with this finding, over 50.0 ± 5.6% (*n* = 3) of cells treated with 5 μ M Tg for 6 h exhibited the whorl phenotype (Figure 1C). In comparison, 11.2 ± 3.3% (*n* = 3) of control cells treated with DMSO exhibited whorl patterns (Figure 1C). Furthermore, Tg treatment at 1 μ M for 6 h induced whorls formation in 33.1% of the treated cells (Figure 1C). Together, these results indicate that ER stress induced by Tg treatment drives whorl formation in a dose-dependent manner.

To check whether ER whorls can be induced by drugs other than Tg, we treated HEK293T cells with several reagents reported in the literature, including MK-28, a PERK activator (Ganz et al., 2020); Palmitic Acid, a long-chain saturated fatty acid (Xu et al., 2020; Harada et al., 2002); Bufalin, a Na^+^/K^+^-ATPase inhibitor (Shen et al., 2014); and CB-5083, a p97 inhibitor (Bastola et al., 2016). These reagents are known to induce ER stress *via* different mechanisms (Ganz et al., 2020; Harada et al., 2002; Shen et al., 2014; Bastola et al., 2016). We generally found them to be less effective than DTT and Tg in inducing ER stress and performed all treatments at 1  μ M for 12 h. Treatment by these reagents all induced formation of ER whorls (Supplementary Figure S2). In addition to these reagents, Cyclopiazonic Acid (CPA) and Lipopolysaccharide (LPS) have also been reported to induce formation of ER whorls in a dose-dependent manner (Xu et al., 2020). Together, these results show that formation of ER whorls is a general hallmark of ER stress rather than a specific outcome of Tg treatment.

Because prolonged ER stress may lead to cell death, we checked whether formation of whorls was caused by cell apoptosis. Using treatment of 10 μ M Etoposide to inhibit DNA replication as a positive control, Propidium Iodide (PI) staining found no cell death under the performed Tg treatment (Figure 1B). Consistent with this result, cleavage of Caspase-3 was not detected by Western blot analysis (Figure 1A). In addition, it was reported that cells treated with Tg for 6 h recovered to exhibit normal ER morphology after Tg was washed out (Xu et al., 2020). Together, these results indicate that formation of whorls results from ER stress rather than cell death.

### Dynamic ER Whorl Formation and Structural Deformation

To examine the dynamic formation of ER whorls, we performed time-lapse live cell imaging. Along with the membrane expansion and aggregation under stress, initial formation of ER whorls started approximately 0.5–1 h after Tg treatment. Severe membrane deformation led to further local ER aggregation (Figure 2B). Over the next 1–5 h, the number of ER whorls continued to increase while existing whorls became more condensed. After 6 h, the number of whorls and their morphologies generally became stable. The whorls occupied most of the intracellular space, and ER network connections were mostly lost (Figure 2B). Quantitative analysis revealed that whorls appeared in ∼21.0% of cells after 1 h treatment (Figure 2C), and the percentage increased over time (Figure 2C). We also examined the three-dimensional structure of whorls (Figure 2D) and found that they were composed of warped ER membranes without ER tubules. Expansion and deformation of ER sheets could contribute to whorl formation. Importantly, canonical ER network connections were largely lost due to absence of ER tubules.

To check the abundance and location of ER proteins on the whorls, we examined fluorescently labeled Rtn4a, ATL3, and receptor accessory protein 5 (REEP5) under induced ER stress. We also checked ER luminal proteins by expressing fluorescently labeled lumen marker KDEL (Supplementary Figure S3A). Overall, Rtn4a rarely locates to ER whorls under Tg treatment. Luminal proteins labeled by KDEL locates to ER whorls in ∼10% of the treated cells, REEP5 locates to ER whorls in ∼20% of the treated cells but ATL3 locates to ER whorls in ∼80% of the treated cells (Supplementary Figure S3B). Overall, these results reveal differential abundance and location of ER proteins on ER whorls.

### Unfolded and Aggregated Proteins are Attached to ER Whorls

**FIGURE 3:**
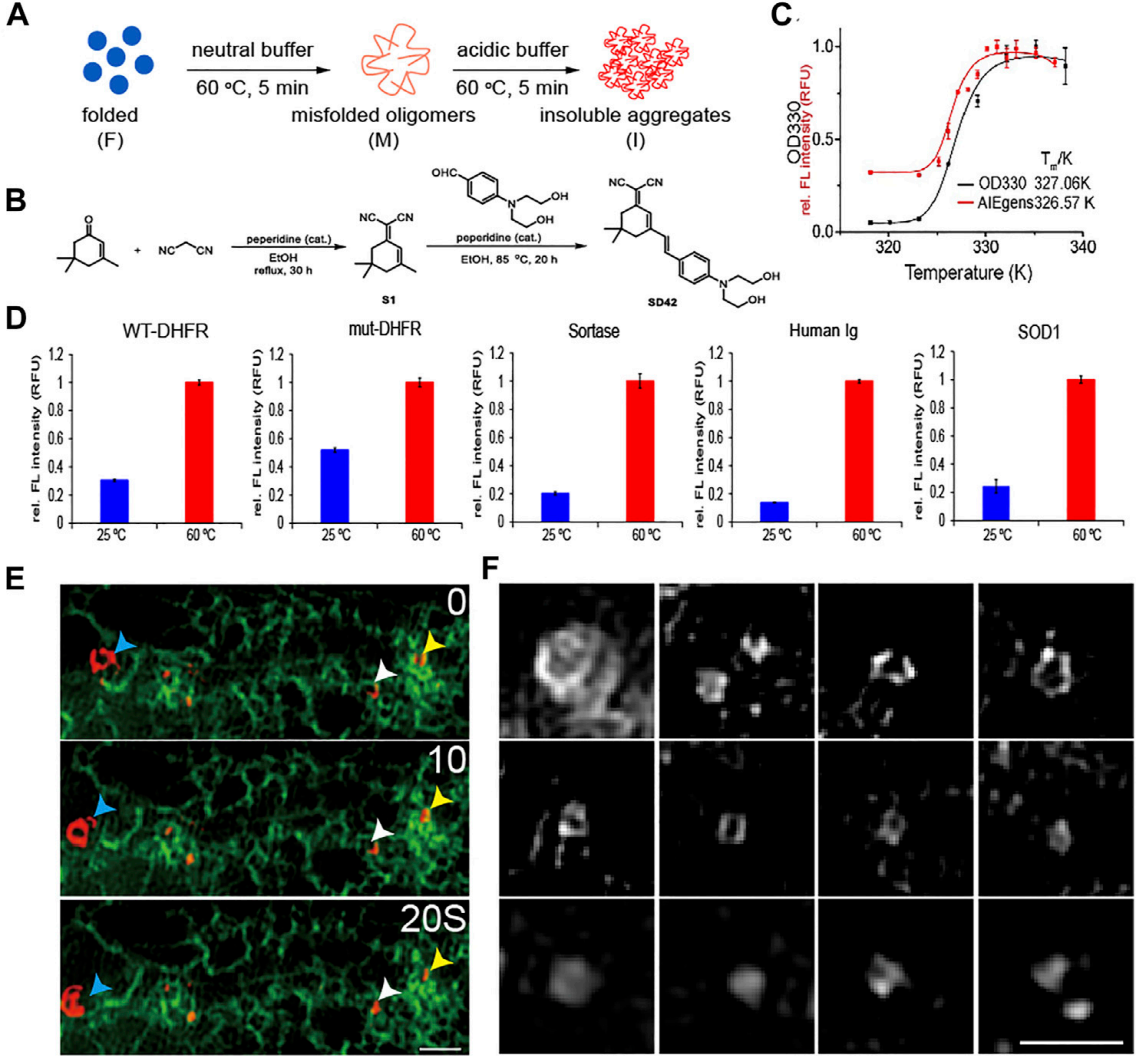
Detecting unfolded and aggregated protein *in vitro* and *in vivo* by AIEgen. **(A)** A cartoon illustration of the formation of misfolded oligomers and insoluble aggregated DHFR model proteins. **(B)** Structural features of the AIEgen. **(C)** Thermal shift assay using OD330 turbidity (black curve) and the fluorescence of the AIEgen (red curve). Fluorescence of AIEgen occurs slightly earlier than the formation of insoluble aggregates measured by OD330 turbidity assay, indicating that the fluorescence originates from misfolded oligomers. **(D)** Fluorescence of AIEgen detecting the folded verses misfolded and aggregated proteins. AIEgen (25 μ M) and WT-DHFR (50 μ M), mut-DHFR (50 μ M), sortase (50 μ M), Human Ig (1 mg/ml) and SOD1 (50 μ M) were mixed in acidic aggregation buffer (NaOAc 200 mM, KCl 100 mM, acidified by AcOH to pH = 6.23) and incubated at 60 °C or 80 °C for 5 min. Spectra were collected with an excitation wavelength of 561 nm. **(E)** Both mobile (blue arrowhead) and immobile (white and yellow arrowheads) unfolded proteins are tightly associated with ER (green). Scale bar = 1 μ m. **(F)** SIM microscopy shows round shapes of unfolded proteins. Scale bar = 1 μ m.

The results so far have revealed tight connections between ER whorl formation and ER stress. Unfolded proteins are a key driver of ER stress (Braakman and Bulleid, 2011). To check whether ER whorls may be used as a reliable image biomarker for ER stress, we examined their relations with unfolded proteins using an AIEgen probe. It exhibited highly specific binding affinity to unfolded and aggregated proteins (Figures 3A–C and Supplementary Figure S4) such as *E. coli* dihydrofolate reductase (DHFR), a model protein for thermal shift assay that detects levels of protein aggregation (Figure 3C). The specific binding affinity of the AIEgen probe was further checked in mut-DHFR, sortase, human immunoglobulin, and superoxide dismutase (SOD1). Relative increase of fluorescence intensity ranging from ∼2 to ∼10 folds was detected, suggesting AIEgen can serve as a general probe for misfolded and aggregated proteins *in vitro* (Figure 3D). With illumination by AIEgen, misfolded and aggregated proteins were detected as puncta *in vivo* in live HEK293T cells and were found to be mobile or immobile (Figure 3E). The puncta were mostly rounded in shape under Structure Illumination Microscopy (SIM), with a diameter of ∼0.5 μ m (Figure 3F).

**FIGURE 4:**
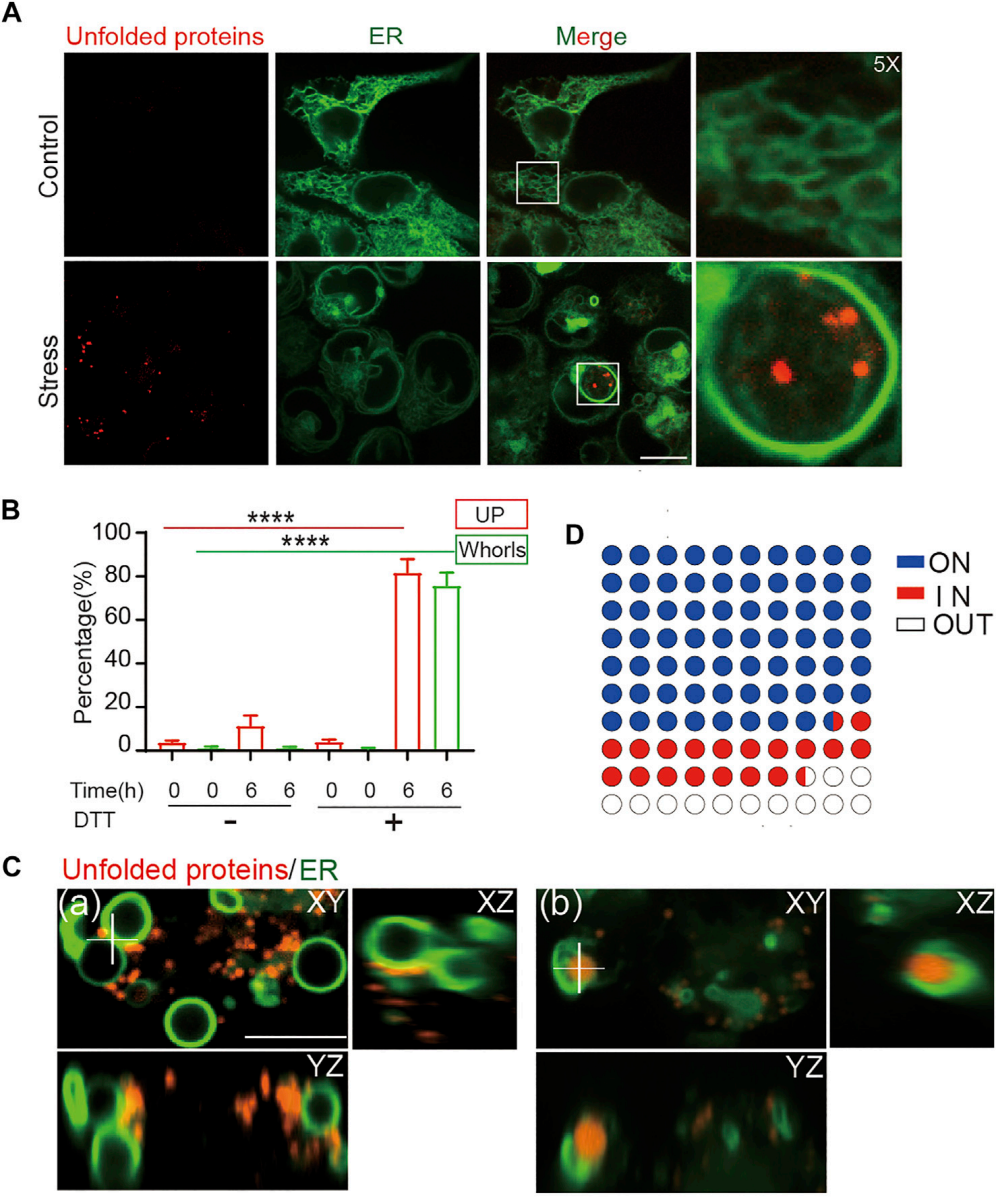
ER whorls tether unfolded proteins under stress. **(A)** ER whorls and unfolded protein (UP) puncta appear only under stress. Scale bar = 10 μm. **(B)** Quantification of unfolded protein puncta and whorls under stress. A 6-h treatment of 10 mM DTT induced both unfolded protein and whorls formation at a final ratio of 81.7 ± 2.1% and 75.7 ± 2.0% (mean ± SD; from n = 235 cells), respectively. ****: *p* < 0.0001. **(C)** 3D view of colocalization of unfolded proteins and whorls. Unfolded proteins may be in contact with the outside surfaces of whorls (a) or exist inside the whorls (b). Scale bar = 1 μm. **(D)** Quantification of localization of unfold proteins with respect to whorls, 68.3 ± 0.3% (mean ± SD; from *n* = 355) of whorls have UPs attached to the membrane (ON), 19.3 ± 0.2% of whorls wrapped UPs inside (IN).

In addition to Tg treatment, ER stress can also be activated by Dithiothreitol (DTT) treatment, which causes protein misfolding and aggregation by blocking formation of disulfide bonds. Similar as under Tg treatment, DTT treatment at 10 mM for 6 h induced formation of ER whorls in 75.7% of treated cells (Figures 4A,B). In contrast, treatment of HEK293T cells with 1 mM of DTT for 6 h only induced formation of ER whorls in a small percentage (4.3%) of cells (Supplementary Figures S5A–C). When the concentration of DTT was increased to 3 mM, formation of ER whorls was detected in 45.3% of treated cells (Supplementary Figures S5A–C), indicating ER stress induced by DTT treatment drives whorl formation in a dose-dependent manner. The rate of whorl formation was significantly lower in control experiments, at 1.2 ± 0.2% (DTT 0 mM, 0 h), 1.2 ± 0.2% (DTT 0 mM, 6 h), and 0.8 ± 0.1% (DTT 10 mM, 0 h). In DTT treated cells, 81.7 ± 2.1% of them showed both puncta of unfolded proteins and ER whorls (Figures 4B,C), significantly higher than that of control cells (11.4 ± 1.6% in DTT 0 mM 6 h, 3.7 ± 0.3% in DTT 0 mM 0 h, and 4.1 ± 0.3% in DTT 10 mM 0 h; *p* < 0.0001; mean ± SEM; *n* = 235 cells from nine experiments). Interestingly, we noticed that the puncta of unfolded protein were tightly associated with deformed ER (Figure 4C). Specifically, 87.5 ± 0.2% of puncta of unfolded proteins were attached to ER whorls during their formation. They were either attached to outer surfaces of the whorls (68.3 ± 0.3%) or were wrapped inside the whorls (19.3 ± 0.2%) (Figures 4C,D) (average ± SEM, *n* = 355 whorls from 151 cells). Taken together, these results indicate that whorls can serve as a morphological image biomarker for ER stress. The tight attachment of unfolded proteins to ER whorls also suggests a direct role of whorls in isolating misfolded and aggregated proteins.

### Development of ER-WHs-Analyzer for Automated Detection of ER Whorls

**FIGURE 5:**
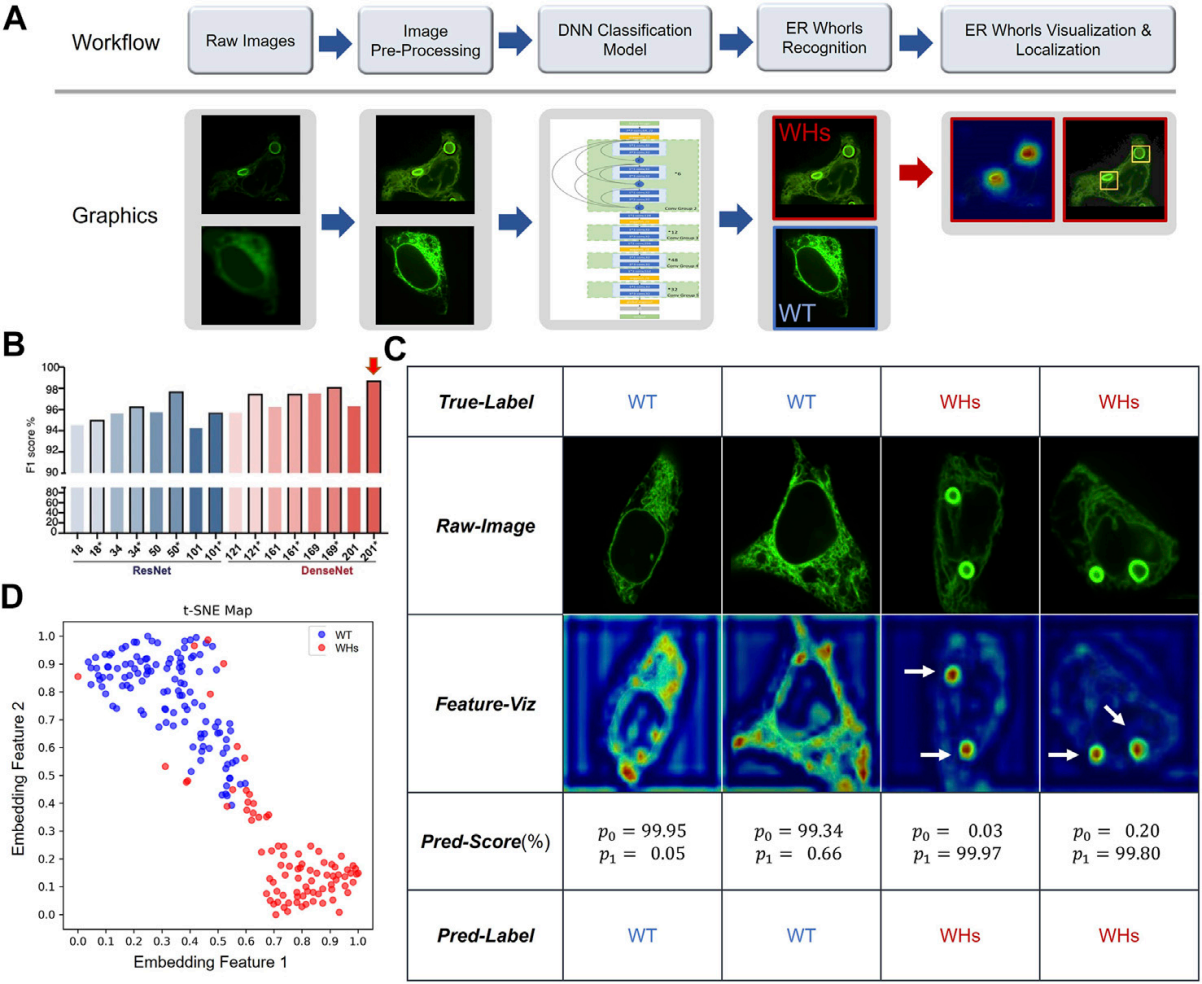
Deep learning-based morphological classification of ER morphology. **(A)** Overall workflow of the proposed ER-WHs-Analyzer. **(B)** Performance evaluation of various DNNs-based classification models in the feature recognition module of ER-WHs-Analyzer. * indicates that the model was finetuned from a pre-trained model. Numbers on *X* axis indicate different depth of the deep learning models. Red arrow indicates the best performed model, the DenseNet-201 which consists of 201 weights-learnable layers. **(C)** Results of images from the ER-Stress-A dataset processed by ER-WHs-Analyzer-v1, including the raw images, their corresponding feature heat maps, their prediction scores, and their predicted labels. **(D)** Based on the phenotypic features learned, a t-SNE map was used to visualize the distribution of wildtype ER images (WT) and the ER images with whorls (WHs).

So far, we have shown that ER whorls can serve as an image biomarker for ER stress. To use it for screening studies, we developed a deep learning-based analysis assay, which we refer to as ER-WHs-Analyzer, for automated detection and analysis of the whorls. The overall workflow of ER-WHs-Analyzer is shown in Figure 5A. First, raw images were cropped from acquired full-size ER images. Then, cropped images were standardized in their sizes and enhanced in their quality through preprocessing. The feature recognition module (DNNs-based classification model) was trained using the preprocessed images along with their binary labels, i.e., WT (wildtype without whorls) or WHs (with whorls) (Figure 5C). The trained feature recognition module was then used to detect whether an ER image contains whorls. If ER whorls were detected, the feature localization module was used to generate a heatmap of features learned by the recognition module. Based on the heatmap, regions of whorls were localized by simple thresholding (Figure 5A; Supplementary Figure S1). Two types of representative DNNs, the ResNet (He et al., 2016) and DenseNet (Huang et al., 2017) (Supplementary Figures S6, S7), were used in this study. Their performance was compared using architectures with different depths including ResNet14, ResNet34, ResNet50, ResNet101, DenseNet121, DenseNet161, DenseNet169 and DenseNet201, where the numbers indicate the count of trainable model layers (Figure 5B; Supplementary Figure S6).

### Training and Testing Deep Learning Models of ER-WHs-Analyzer

For training and testing of the feature recognition module in ER-WHs-Analyzer, we constructed a dataset that we referred to as ER-Stress-A. We collected 490 cell images of normal ER morphology (labeled as WT) or abnormal ER morphology with whorls (labeled as WHs). We split the images into a training set, a validation set, and a test set (Supplementary Table S1). The training and validation sets were used to finetune training hyper-parameters (Supplementary Table S2). The test set was used for standalone testing. Standard performance metrics for image classification were used, including F1 (F1 score), AUC (area under ROC curve), ACC (accuracy), Spc (specificity), Sen (sensitivity), Pre (precision). The validation set in ER-Stress-A was used to compare performance of different architectural configurations of backbone networks.

Validation results of different configurations of backbone networks are compared in Figure 5B. Several observations can be made. First, deeper networks generally provided better performance. For example, ResNet50 with 50 weight-learnable layers obtained a higher F1-score than ResNet34 and ResNet18. And DenseNet generally outperformed ResNet. Second, models pretrained with CBMI-Extra (indicated by *) generally outperformed models without pretraining. The benefit of pretraining was more pronounced for shallow models. For example, the ResNet34 pretrained with CBMI-Extra outperformed DenseNet121 without pretraining. Third, deeper networks such as ResNet101 and DenseNet201 were more prone to overfitting. This problem can be mitigated by finetuning. DenseNet 201 achieves overall the highest rate of recognizing ER whorls (98.78%) (Figure 5B). Other performance metrics are listed in Supplementary Table S5. We chose DenseNet201 with pretraining for the feature recognition module of ER-WHs-Analyzer. In standalone testing, the model achieved excellent performance with F1 = 98.27%, AUC = 99.65%, ACC = 98.54%, Spc = 99.16%, Sen = 97.70%, Pre = 98.84%. In model testing, the ratio of abnormal ER structures labeled by experts was 42.33%, while the ratio detected by ER-WHs-Analyzer is 42.16%, reaching a high level of agreement. After feature recognition, the feature localization module of ER-WHs-Analyzer determined positions of the regions of whorls based on a heatmap of learned feature and subsequent thresholding (Figure 5A).

Representative images from ER-Stress-A test set and their correct classification labels are shown in the second row and the first row of Figure 5C, respectively. The heatmaps of learning features are shown in the third row of Figure 5C, while their recognition results using the feature recognition module are shown in the fourth and fifth rows, respectively. Together, the results showed that ER-WHs-Analyzer can accurately recognize and localize individual ER whorls (Figure 5C; Supplementary Figure S8). We also used a t-SNE map (Maaten and Hinton, 2008) to visualize the representative features that our method learned to separate morphology of normal ER from ER with whorls (Figure 5D). We found that morphology of ER under normal condition and induced stress can be well differentiated.

### Separating Different Sub-Phenotypes of ER Whorls Using ER-WHs-Analyzer

**FIGURE 6:**
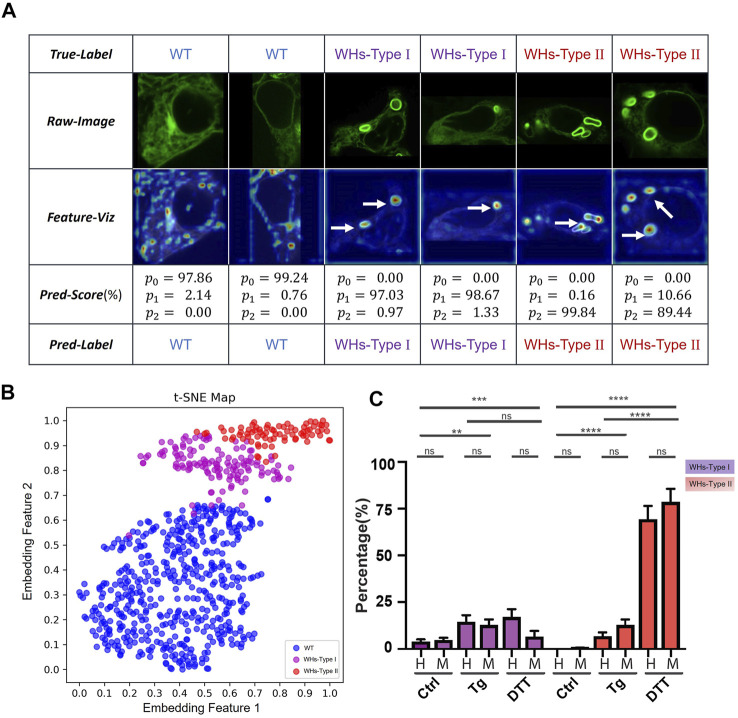
Two sub-phenotypes of ER whorls were identified by ER-WHs-Analyzer-v2. **(A)** Results of images from the ER-Stress-B dataset processed by ER-WHs-Analyzer-v2, including the raw images, their corresponding feature heat maps, their prediction scores, and their predicted labels. **(B)** Based on the phenotypic features learned, a t-SNE map was used to visualize the distribution of wildtype ER images (WT), the ER images with WHs-Type I, and the ER images with WHs-Type II. **(C)** Double-blind testing results between human experts (H) and ER-WHs-Analyzer v2 (M). Purple bars show the distribution of WHs-Type I. Red bars show the distribution of WHs-Type II. Error bars indicate SEM, which was computed from 81 sampled images. **: *p* < 0.01; ***: *p* < 0.001; ****: *p* < 0.0001, ns: not significant.

ER stress is induced by treatment of Tg and DTT *via* different mechanisms (Dibdiakova et al., 2019). Tg induces ER stress *via* interfering with calcium ion transport, while DTT induces ER stress by blocking the formation of disulfide bonds required for protein folding (Jiang et al., 2015). Differently from Tg or Tunicamycin (Tm), DTT is also considered as a robust pro-apoptotic ER stress inducer (Labunskyy et al., 2009). As expected, DTT treatment at a concentration of 3 mM caused formation of ER whorls. However, ER whorls induced by DTT (Figure 6A, second row, panels 5–6) showed morphological difference from those induced by Tg treatment (Figure 6A, second row, panels 3–4). Specifically, whorls induced by Tg treatment generally tend to be circular and small whereas whorls induced by DTT treatment tend to be elliptic and large. Cells treated with Tg generally tend to have a low number of whorls, typically one or two. Cells treated with DTT generally tend to have more whorls, typically two or more. We quantitatively analyzed the number and area of whorls in the cells treated by Tg and DTT using feature localization module (Supplementary Figure S1). Overall, DTT treatment induced an average of 2.43 ± 1.36 whorls per cell (mean ± SD; *n* = 112 cells) and an average area of whorls of 29.27 ± 16.75 $\mu m^{2}$ (*n* = 272 whorls). In contrast, Tg treatment induced an average of 1.37 ± 0.80 whorls per cell (*n* = 150 cells) and an average area of 14.88 ± 11.79 $\mu m^{2}$ (*n* = 206 whorls). The differences in whorl number and area between the two treatments are statistically significant (*p* < 0.0001).

To verify whether our ER-WHs-Analyzer can separate these morphological subphenotypes of ER whorls, we constructed another dataset that we refer to as ER-Stress-B. It contains three categories: Wildtype, WHs-Type I and WHs-Type II. WHs-Type I contains one or two whorls and WHs-Type II contains more than two whorls. ER-Stress-B contains a total of 1404 cell images. We partitioned all the images into a training set and a validation set (Supplementary Table S3). We used the same deep learning model and performance metrics as in previous experiments. For this multi-class recognition task, we replaced the previous two-class output layer with a three-class output layer and retrained the models. Again, we compared the performance of our models with and without pretraining with the CBMI-Extra dataset (Supplementary Table S4). From the results, we observed a similar trend as in previous two-class classification experiments. Overall, DenseNet outperforms ResNet, and models with pretraining (indicated by *) outperform those without. However, differently from previous experiments, DenseNet161 shows overall the best performance, whose F1 score reaches 98.86%. Detailed evaluation metrics are compared in Supplementary Table S6. We chose DenseNet161 with pretraining for the feature recognition module and referred to the overall assay as ER-WHs-Analyzer v2.

Next, we checked whether ER-WHs-Analyzer v2 can reliably separate different subphenotypes of ER whorls. Figure 6A shows the results, which confirms that ER morphologies of WHs-Type I and WHs-Type II can be reliably differentiated. The t-SNE map (Figure 6B) shows that ER-WHs-Analyzer v2 has learned representative features to distinguish the sub-phenotypes of ER whorls.

Finally, we performed a double-blind experiment to compare ER-WHs-Analyzer v2 against human experts in classifying ER images acquired under control, Tg treatment, and DTT treatment respectively. Based on the classification results, we counted the numbers of the cells belonging to different sub-phenotypes for the control group and the two experimental groups. From the results, we have observed clear distribution differences of these three groups of cells (Figure 6C). According to classification by human experts, the control group contains 96.1 ± 7.3% WT, 3.9 ± 1.2% WHs-Type I and 0.0 ± 0.0% WHs-Type II. The Tg treatment group contains more WHs-Type I, with 78.7 ± 4.8% WT, 14.6 ± 3.5% WHs-Type I, and 6.8 ± 2.1% WHs-Type II (Figure 6C). In contrast, the DTT treatment group contains more WHs-Type II, with 13.6 ± 6.8% WT, 17.0 ± 4.2% WHs-Type I, 69.3 ± 7.1% WHs-Type II (Figure 6C). Classification results by ER-WHs-Analyzer v2 generally matched those by human experts (*p* > 0.07, n > 81 from seven experiments) (Figure 6C). Together, these experimental results suggested that ER-WHs-Analyzer v2 can reliably detect morphological differences of ER whorls induced by different treatments.

## Discussion

In this study, we examined morphological patterns of ER under stress and identified ER whorls as an image biomarker of ER stress for screening studies. ER whorls are found in both yeast and mammalian cells, and their formation is considered as an integral part of cellular response to ER stress (Bernales et al., 2006; Schäfer et al., 2020; Xu et al., 2020), an important target of drug development studies for treatment of cancer as well as metabolic and neurodegenerative diseases. Although a wide variety of chemical and genetic tools for assessing ER stress have been developed (Sicari et al., 2020), image biomarkers for efficient phenotypic screening have been lacking. Our study fills this gap.

Monitoring ER stress by detecting and analyzing ER whorls carry some important advantages. Canonical ER stress detection is carried out by Western blot analysis of the expression or phosphorylation of ER stress modulators or their transcription by quantitative reverse transcription polymerase chain reaction (RT-PCR) (Haynes and Wiseman, 2018). These are endpoint assays that are laborious. More importantly, they cannot measure ER stress in single cells. Another approach to detect ER stress is to monitor the transcription reporters of molecules key to UPR, such as XBP1 and ATF4, by fluorescence. But it is not a real-time approach because of the time delay between initiation of transcription and the illumination of reporter proteins. In comparison, our study shows that ER morphological changes correlate well ER stress, and ER whorls can be used as an image biomarker to detect ER stress. They appear as early as 1 h after stress induction, indicating that they respond quickly to ER stress. Furthermore, high-resolution live-cell imaging of whorls makes real-time and single-cell level monitoring of ER stress possible. Our study combines automated high-resolution ER microscopy with deep learning-based analysis using ER-WHs-Analyzer to achieve high-throughput observation and quantification. This automated monitoring and analysis assay can be used as an effective tool for screening or validation of ER stress-related targets or drugs. It can reliably separate different sub-phenotypes of ER morphology under stress induced by different drugs. It can also be trained for use with other specific image biomarkers for ER stress. However, experimental settings of our study such as concentration and duration of Tg and DTT treatment remain be further optimized.

The mechanism regulating ER whorl formation is complicated. Although the causal relation between formation of ER whorls and COPII, ESCRT has been established, the relation between formation of ER whorls and PERK signaling remains unclear (Schäfer et al., 2020; Xu et al., 2020). Our study shows that formation of ER whorls accompanies activation of IRE1 α . Therefore, multiple signaling pathways likely contribute to whorl formation. The precise functions of ER whorls are not completely clear either. The oligomerization and *trans*-autophosphorylation are considered as an activation of ER stress sensors. Therefore, whether whorls contribute to the activation of PERK and IRE1 α  should be tested. In addition, ER whorls are reported to separate the translocon complex and suppress protein translation (Xu et al., 2020). We have also found that they isolate misfolded and aggregated proteins. However, whether they serve other cellular functions and whether they contribute to cell fate determination under prolonged ER stress remained to be determined. The automated image biomarker assay developed in this study will help address these questions.

## Data Availability

The original contributions presented in the study are included in the article/Supplementary Material, further inquiries can be directed to the corresponding authors.
