# Supplementary material for: Deep Learning-Based Morphological Classification of Endoplasmic Reticulum Under Stress
Source: Front Cell Dev Biol. 2022 Jan 21;9:767866. doi: 10.3389/fcell.2021.767866 (PMC8865080; doi:10.3389/fcell.2021.767866)
Supplement: Supplementary file 1 [file DataSheet1.PDF]

# *Supplementary Materials*

## Deep Learning-Based Morphological Classification of Endoplasmic Reticulum Under Stress

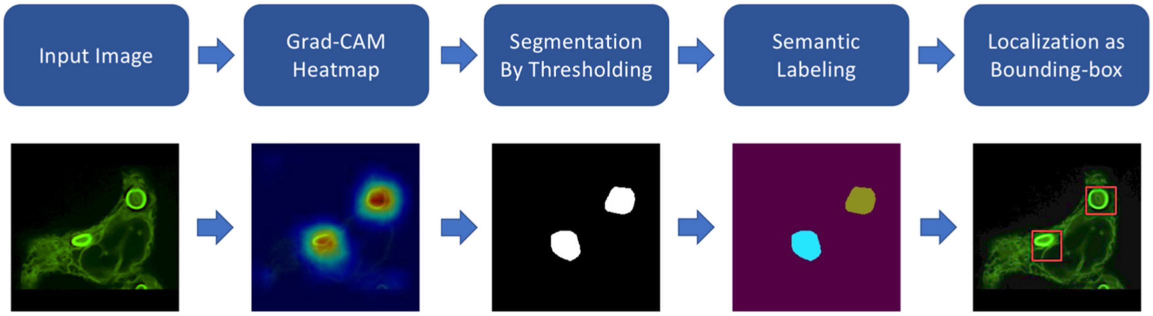


**Fig. S1. Image processing pipeline of the feature localization module in ER-WHs-Analyzer.** The pipeline takes heatmaps of learned features generated by Grad-CAM as its input and uses a simple thresholding-based strategy to localize regions of ER whorls. Orange boxes indicate estimated locations of localized regions of whorls.

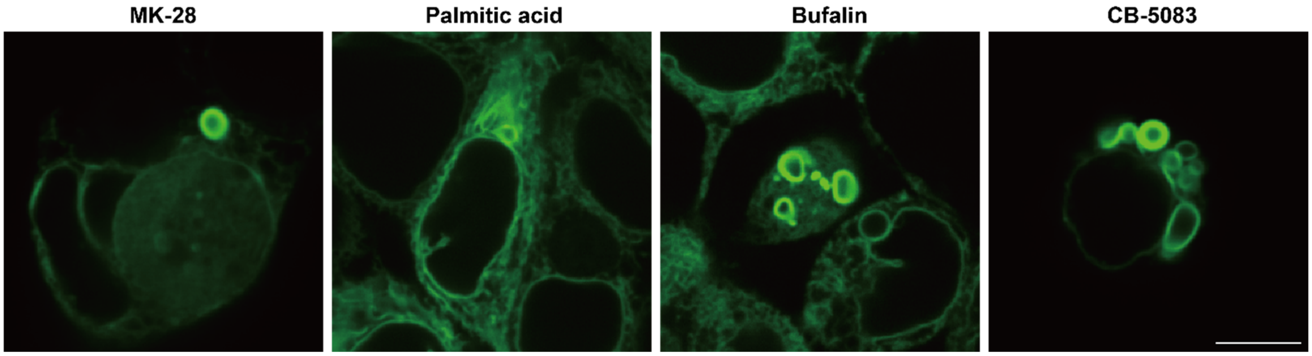


**Fig. S2. Formation of ER whorls under treatment of different ER stress inducers.** ER whorls were observed in HEK293T cells treated with MK-28, Palmitic acid, Bufalin, and CB-5083 at 1 $\mu$M for 12 hours. Scale bar: 10 µm.

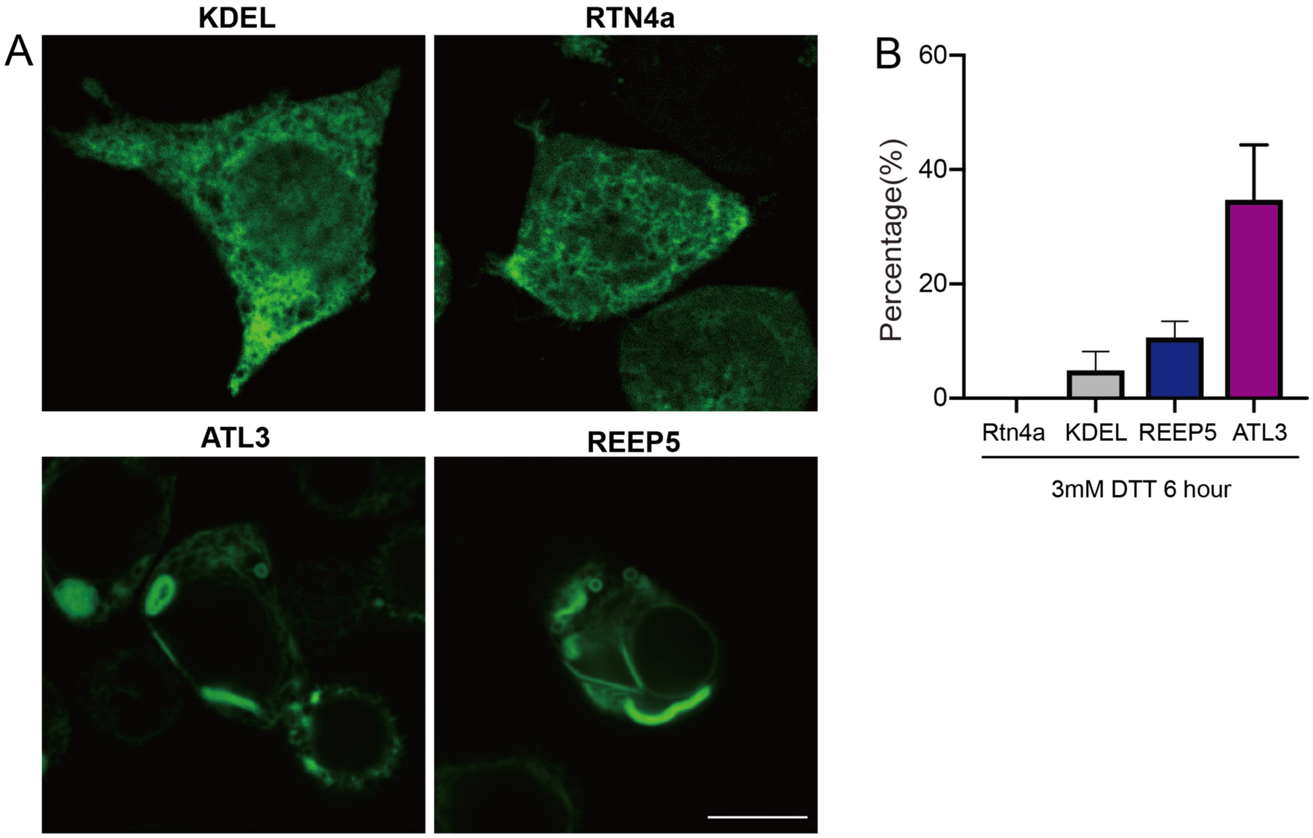


**Fig. S3. Abundance and location of ER proteins on whorls.** (A) ER morphology in the HEK293T cells treated with 3 mM DTT for 6 hours and expressing fluorescently labeled BFP-KDEL, mCherry-Rtn4a, mCherry-ATL3, and GFP-REEP5. (B) Percentage HEK293T cells with curvature proteins locating to ER whorls. Data were presented as mean ± standard error (SD) for each protein from > 41 cells in 3 independent experiments.

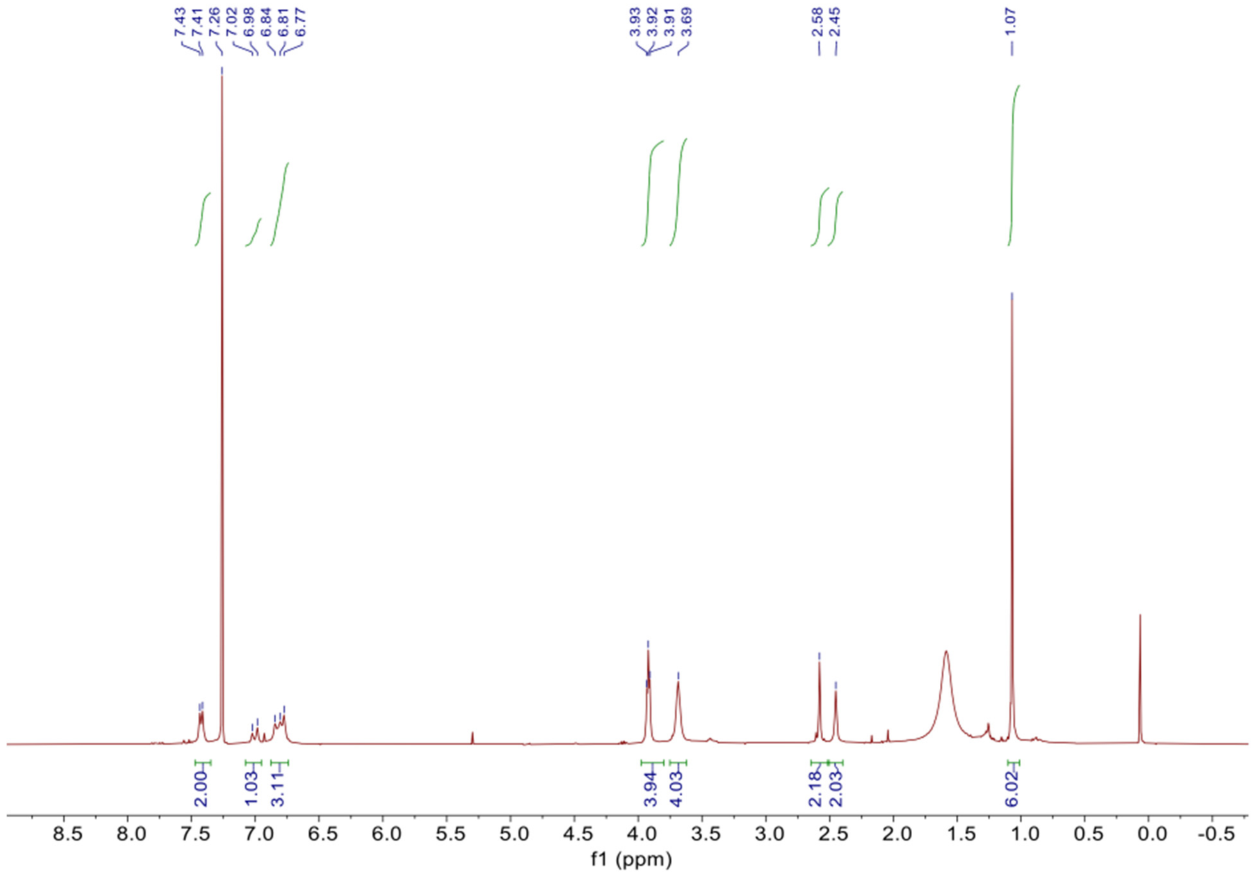

**Fig. S4. Synthesis and validation of AIEgen probe for detection of misfolded and aggregated proteins.** NMR analysis of AIEgens. $^{1}H$-NMR (400 MHz, $CDCl3$) δ 7.42 (m, 2H), 7.00 (m, 1H), 6.88 – 6.74 (m, 3H), 3.92 (t, $J$ = 4.9 Hz, 4H), 3.69 (s, 4H), 2.58 (s, 2H), 2.45 (s, 2H), 1.07 (s, 6H). HRMS (m/z) Anal. Calc'd for $C_{23}H_{28}N_3O_2$ $(M+H)^+$ : 378.2716, Found $(M+H)^+$ : 378.2175.

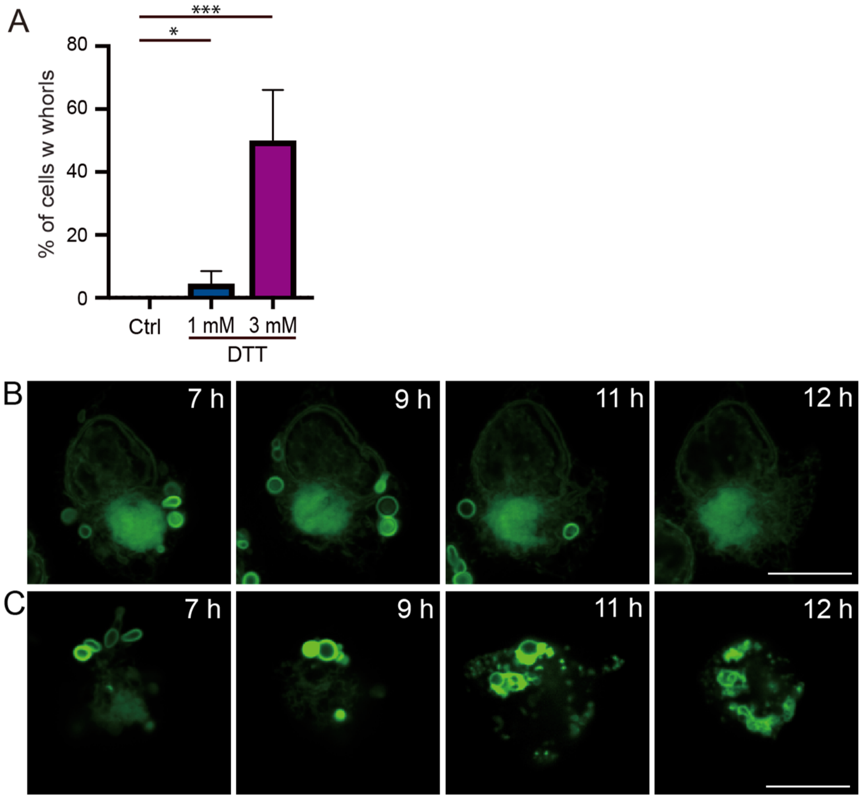


**Fig. S5. Cell fates after treatment of 3mM DTT for 6 hours.** ER structures labeled with GFP-Sec61$\beta$ (green) in HEK293T cells after treatment with 3 mM DTT for 6 hours an. (A) Percentage of HEK293T cells with ER whorls under control condition or treatment with DTT for 6 hours. Error bars indicate standard deviation (SD) calculated from 3 independent experiments. *: $p < 0.05$, **: $p < 0.01$; ***: $p < 0.001$. (B) In vast majority of the cells, ER whorls remain stable. (C) A small number of cells entered apoptosis. Scale bars: 10 $\mu$m.

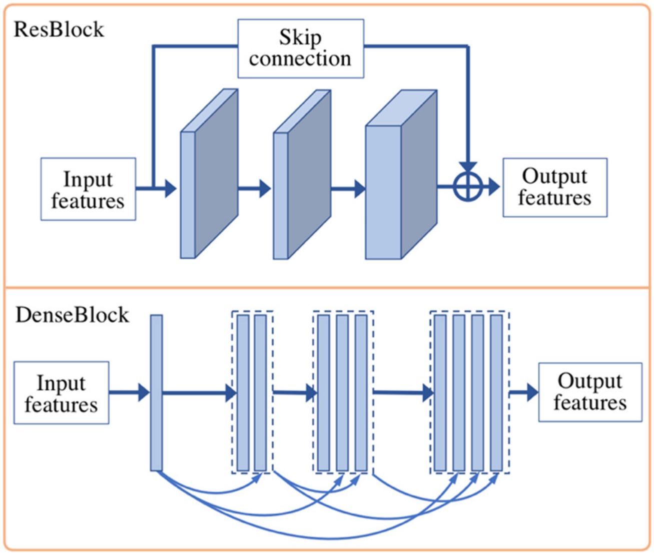


**Fig. S6. Basic building blocks of ResNet and DenseNet.** The ResBlock consists of a cascade of convolution layers with different kernel sizes and numbers of channels. The skip connection, which directly adds the input to the output, is used to facilitate training of deep neural networks. The DenseBlock concatenates all previous features into the current convolution layer. This type of skip connection links features over a long range and mitigates the problems of vanishing/exploding gradient.

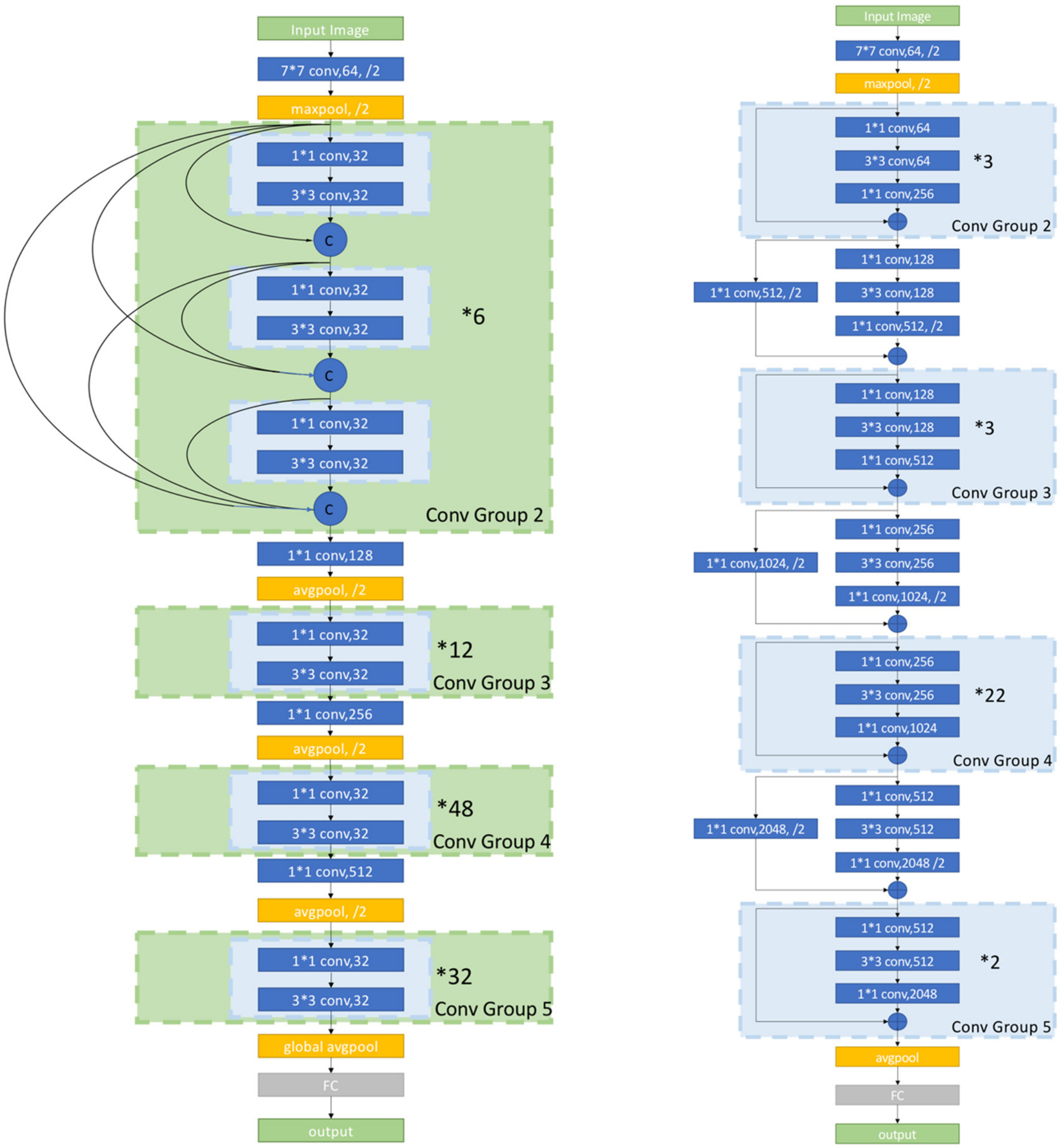


(A) DenseNet201 (B) ResNet101

**Fig. S7. Network architectures of (A) DenseNet201 and (B) ResNet101.** Both networks start with an input convolution layer with a large kernel size, i.e. 7×7, followed by a downsampling layer. The green dashed block in (A) and blue dashed block in (B) correspond to the DenseBlock and ResBlock in Figure S2, respectively. The numbers in each convolution group indicate the numbers of repetitions of corresponding blocks. Other configurations of DenseNet and ResNet, e.g., DenseNet161 and ResNet50, can be obtained by repeating the DenseBlock and ResBlock in each convolution group for corresponding numbers of times. Convolution layers between neighboring convolution groups in ResNet101 are transition layers to downsample feature maps.

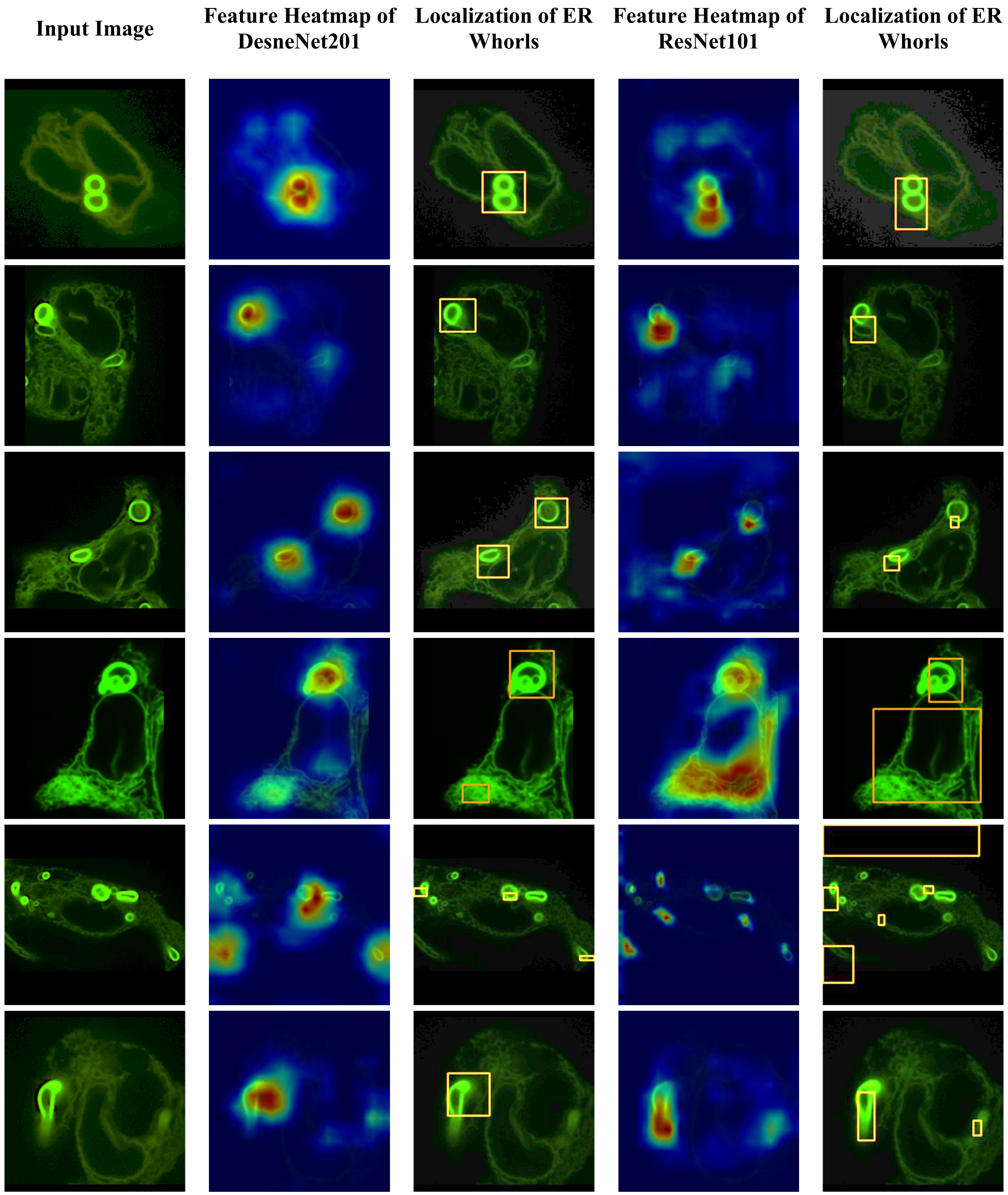


**Fig. S8. Results of automated localization of regions of ER whorls by the feature localization module of ER-WHs-Analyzer.** Localized regions of whorls are indicated by orange boxes. DenseNet generally learns more discriminative features than ResNet to localize regions of whorls, consistent with their performance in classifying these patterns.

**Table S1: Statistics of dataset ER-Stress-A**

| Split | WT | WHs | Total (490) |
|---|---|---|---|
| Training Set | 59 | 83 | 142 |
| Validation Set | 60 | 82 | 142 |
| Test Set | 119 | 87 | 206 |

**Table S2: Training configurations on ER-Stress-A**

| Networks | Pretrained | Learning Rate | Batch Size | Epochs | Momentum | Learning Rete Decay |
|---|---|---|---|---|---|---|
| ResNet / DenseNet | True | 0.005 | 32 | 400 | 0.9 | *0.8 for every 30 epoch |
| | False | 0.01 | 16 | 600 | 0.9 | *0.8 for every 40 epochs |

**Table S3: Statistics of dataset ER-Stress-B**

| Split | WT | WHs-Type I | WHs-Type II | Total (1404) |
|---|---|---|---|---|
| Training Set | 494 | 130 | 79 | 703 |
| Validation Set | 494 | 129 | 78 | 701 |

**Table S4: Training configurations on ER-Stress-B**

| Networks | Pretrained | Learning Rate | Batch Size | Epochs | Momentum | Learning Rete Decay |
|---|---|---|---|---|---|---|
| ResNet / DenseNet | True | 0.005 | 32 | 400 | 0.9 | *0.8 for every 30 epoch |
| | False | 0.01 | 16 | 600 | 0.9 | *0.8 for every 40 epochs |

**Table S5: Performance of different models in classifying ER whorls.**

| Networks | F1 (%) | AUC (%) | ACC (%) | Spc (%) | Sen (%) | Pre (%) |
|---|---|---|---|---|---|---|
| ResNet18 | 94.55 | 97.34 | 93.66 | 91.67 | 95.12 | 93.98 |
| ResNet18* | 95.06 | 97.52 | 94.37 | 95.00 | 93.90 | 96.25 |
| ResNet34 | 95.65 | 98.48 | 95.07 | 96.67 | 93.90 | 97.47 |
| ResNet34* | 96.34 | 98.41 | 95.77 | 95.00 | 96.34 | 96.34 |
| ResNet50 | 95.76 | 98.27 | 95.07 | 93.33 | 96.34 | 95.18 |
| ResNet50* | 97.76 | 97.50 | 97.18 | 96.67 | 97.56 | 97.56 |
| ResNet101 | 94.27 | 97.05 | 93.66 | 98.33 | 90.24 | 98.67 |
| ResNet101* | 95.76 | 98.27 | 95.07 | 93.33 | 96.34 | 95.18 |
| DenseNet121 | 95.71 | 98.01 | 95.07 | 95.00 | 95.12 | 96.30 |
| DenseNet121* | 97.53 | 99.11 | 97.18 | 98.33 | 96.34 | 98.75 |
| DenseNet161 | 96.25 | 97.58 | 95.77 | 98.33 | 93.90 | 98.72 |
| DenseNet161* | 97.53 | 99.17 | 97.18 | 98.33 | 96.34 | 98.75 |
| DenseNet169 | 97.53 | 97.32 | 97.18 | 98.33 | 96.34 | 98.75 |
| DenseNet169* | 98.18 | **99.65** | 97.89 | 96.67 | 98.78 | 97.59 |
| DenseNet201 | 96.34 | 98.13 | 95.77 | 95.00 | 96.34 | 96.34 |
| DenseNet201* | **98.78** | 99.15 | **98.59** | **98.33** | **98.78** | **98.78** |

* Models pre-trained CBMI-Extra.
Bold numbers indicate best results.

**Table S6: Performance of different models in separating ER morphological subphenotypes.**

| Networks | F1 (%) | AUC (%) | ACC (%) | Spc (%) | Sen (%) | Pre (%) |
|---|---|---|---|---|---|---|
| ResNet18 | 97.70 | 99.86 | 95.38 | 97.72 | 97.72 | 97.70 |
| ResNet18* | 98.14 | 99.80 | 95.84 | 98.15 | 98.15 | 98.18 |
| ResNet34 | 97.85 | 99.87 | 95.64 | 97.86 | 97.86 | 97.86 |
| ResNet34* | 98.42 | 99.87 | 96.31 | 98.43 | 98.43 | 98.42 |
| ResNet50 | 97.12 | 99.68 | 93.99 | 97.15 | 97.15 | 97.16 |
| ResNet50* | 97.71 | 99.78 | 95.23 | 97.72 | 97.72 | 97.70 |
| ResNet101 | 96.45 | 99.80 | 94.01 | 96.43 | 96.43 | 96.53 |
| ResNet101* | 97.02 | 99.28 | 95.07 | 97.00 | 97.00 | 97.05 |
| DenseNet121 | 98.00 | 99.89 | 95.73 | 98.00 | 98.00 | 97.99 |
| DenseNet121* | 98.71 | 99.92 | 97.34 | 98.72 | 98.72 | 98.72 |
| DenseNet161 | 98.15 | 99.90 | 96.68 | 98.15 | 98.15 | 98.18 |
| DenseNet161* | **98.86** | 99.93 | **98.15** | **98.86** | **98.86** | **98.86** |
| DenseNet169 | 98.14 | 99.84 | 96.13 | 98.15 | 98.15 | 98.17 |
| DenseNet169* | 98.71 | **99.94** | 97.34 | 98.72 | 98.72 | 98.72 |
| DenseNet201 | 98.01 | 99.90 | 95.75 | 98.00 | 98.00 | 98.01 |
| DenseNet201* | 98.57 | 99.90 | 96.91 | 98.57 | 98.57 | 98.57 |

* Models pre-trained CBMI-Extra.
Bold numbers indicate best results.
